# Identification of a Novel Enterovirus E Isolates HY12 from Cattle with Severe Respiratory and Enteric Diseases

**DOI:** 10.1371/journal.pone.0097730

**Published:** 2014-05-15

**Authors:** Lisai Zhu, Zeli Xing, Xiaochun Gai, Sujing Li, Zhihao San, Xinping Wang

**Affiliations:** College of Veterinary Medicine, Jilin University, Changchun, Jilin, China; University of Illinois at Chicago, United States of America

## Abstract

In this study, a virus strain designated as HY12 was isolated from cattle with a disease of high morbidity and mortality in Jilin province. Biological and physiochemical properties showed that HY12 isolates is cytopathic with an extremely high infectivity. HY12 is resistant to treatment of organic solvent and acid, and unstable at 60°C for 1 h. Electron microscopy observation revealed the virus is an approximately 22–28 nm in diameter. The complete genome sequence of HY12 consists of 7416 nucleotides, with a typical picornavirus genome organization including a 5′-untranslated region (UTR), a large single ORF encoding a polyprotein of 2176 amino acids, and a 3′-UTR. Phylogenetic analysis clustered HY12 isolates to a new serotype/genotype within the clade of enterovirus E (formerly BEV-A). Alignment analysis revealed a unique insertion of 2 amino acid residues (NF) at the C-terminal of VP1 protein between aa 825 and 826, and several rare mutations in VP1 and VP4 of HY12 isolates in relation to known bovine enterovirus (BEV) strains. This is the first report of an enterovirus E in China, which is potentially associated with an outbreak in cattle with severe respiratory and enteric diseases.

## Introduction

The genus *enterovirus* within the family *picornaviridae* consists of 9 species of enterovirus (A,B, C, D, E, F, G, H, J) and 3 species of rhinoviruses (A, B, C) based on the latest virus taxonomy[1]. These viruses have many features in common and are the leading etiological agents related to respiratory and digestive diseases in human and animals. Like other species within the genus, bovine enteroviruses (BEVs) are small, non-enveloped viruses with an icosahedral virion and a positive–stranded RNA genome. BEVs have been isolated from cattle with a clinical signs varying from respiratory diseases to enteric, reproductive disease and infertility [2], [3], [4], [5], [6], [7], [8], [9], [10], [11], [12], [13], [14], and even from faeces of the presumably healthy calves[15]. The pathogenicity and virulence of bovine enterovirus is still largely unknown. Failure to experimentally reproduce bovine enterovirus infection in calves showing obvious clinical signs led to the conclusion that BEV is not significant agent in the cattle industry. This argument was seemly further supported by the findings that bovine enterovirus were easily detected in the contaminated waters adjacent to cattle herds; and the discovery that BEV-like sequences are present in shellfish, bottlenose dolphins, and in deer feces from the same geographical area [16], [17], [18], [19], [20]. However, with more and more BEV isolates were identified from the fatal enteric and respiratory disease, the pathogenicity and virulence of BEV recurred where its relatedness to the illness be intensively explored [12], [21], [22], [23], [24]. Blas-Machdo has showed that calves experimentally infected with BEV-1 manifested symptoms of respiratory illness similar to those naturally BEV-infected calves; and the BEV-1 was detected to localize mainly in the digestive tract, indicating the potential pathogenicity of BEV [22]. Recently, several strains of BEV were isolated from cattle with a severe diarrhea in China, and genomic sequence analysis indicated that those strains belong to enterovirus F (previously named BEV-B)[12]. Those findings suggest the pathogenicity of BEV as potential causative agents for the respiratory and enteric diseases in cattle.

Classification of bovine enteroviruses undergoes a series of modifications. In the early attempt, BEV were classified into seven serotypes, and later revised to two serotypes [25], [26]. Because of the cross-reaction among BEV type-specific sera, it is difficult to type BEV using serological means. With the accumulation of BEV sequence data, classification of BEVs based on virus genetic variability and molecular difference becomes feasible. Based on the generally accepted definition for picornavirus species and serotype, a molecular-based BEV classification by comparing the sequences from 5′-UTR, the capsid protein region were established and used to classify bovine enteroviruses to BEV-A (currently named enterovirus E) and BEV-B (currently named enterovirus F), where different serotypes/genotypes were further divided for either enterovirus E or enterovirus F [27], [28], [29]. In this study, we reported the identification of a novel enterovirus E isolates HY12 from a cattle herd with an outbreak of a severe respiratory disease and enteritis in Jilin Province.

## Materials and Methods

The ethics committee in Jilin University has approved this study.

### Cell culture and virus isolation

Faecal samples were collected from diarrheic cattle following standard procedures approved by the ethics committee of College of Veterinary Medicine at Jilin University, and processed to inoculate the MDBK cells. Briefly, samples were diluted in a dilution of 1∶10 (W/V) with 10 mM phosphate buffered saline (PBS) (pH 7.2). After centrifugation at 8000 r. p. m. for 30 min, the supernatant was filtered with 0.45 nm filter and the flow-through was used to inoculate MDBK cells. The inoculum was discarded after incubation with MDBK cells for 2 h, and the cells were washed with Hank's solution before the addition of Dulbecco's modified eagle's medium (DMEM) (Invitrogen,) supplemented with 2% fetal bovine serum (HyClone), 2 µg/ml gentamycin and 2 mM L-glutamine (Invitrogen). The cells were observed every 4∼6 h and cytopathic effects (CPE) were captured using Digital Camera.

### Electron microscopy observation

Sample was processed for EM observation by centrifuging at 8000 r. p. m for 30 min at 4°C after virus cultures were frozen and thawed for 3 times. The supernatants were incubated with 1% phosphotungistic acid, and viruses were observed using electron microscopy (JEM-2200FS/CR).

### TCID_50_ titration and characterization of isolates HY12

Titration of TCID_50_ for HY12 isolates was performed using 96 well-plates. Briefly, viruses were diluted at a 10×serial dilutions and used to infect the quadruplicate wells for each dilution. 48 h post inoculation, the cytopathic effects were observed and counted, and the TCID_50_ was calculated following a standard procedure[30]. Biological and physiochemical properties of HY12 were characterized according to the standard protocols. HY12 viruses were treated either with organic solvents (chloroform and ether), heated at 50°C, 60°C, 70°C, and 80°C for 1 h, or incubated at pH 3.0 and pH 5.0 before infecting the cells. The stability to heat, acid or organic solvents were determined by comparing the TCID_50_ for treated groups and untreated controls.

### RNA isolation, cDNA synthesis and PCR amplification

RNA isolation was performed as previously reported[31]. Briefly, the infected cells were lysed in TRNzol reagent (Tiangen, Beijing), then mixed with 0.2 volume of chloroform and shaken vigorously for 30 sec. After centrifugation at 12,000×g for 20 min at 4°C, the aqueous phase was mixed with equal volume of isopropanol, centrifuged at 12,000×g for 20 min at 4°C. The pellet was washed with 70% ethanol and dissolved in DEPC-treated H_2_O. The resulting RNA was kept at −80°C for further analysis.

The reverse transcriptase reaction was performed using Superscript ™ II Reverse Transcriptase (Invitrogen, Carlsbad, CA). Briefly, cDNA was synthesized in a volume of 20 µl containing 25 mM Tris-HCI, pH 8.3, 37.5 mM KCI, 1.5 mM MgCI2, 5 mM DTT, 0.25 mM each of dATP, dCTP, dGTP and dTTP, 40 units of RNase inhibitor, 200 units of M-MLV reverse transcriptase, 2 µg of total RNA, and 2.5 µM random primers. The cDNA synthesis was carried out at 42°C for 1 h.

PCR amplification was done using Taq DNA polymerase (Takara Bio Group). The reaction was performed in a total volume of 50 µl containing 20 mM Tris-HCI, pH 8.4, 50 mM KCI, 3 mM MgCI_2_, 0.25 mM each of dATP, dCTP, dGTP and dTTP, 5 unit of Taq DNA polymerase, 1 µM of each primer, and 2 µl of cDNA synthesized above. The amplification was done after optimizing the condition. The primers used for amplifying the potential virus sequences were listed in table 1; and primers to amplify the complete genome sequence of HY12 isolates were listed in table 2.

**Table 1:** Primers used for differentiation of the potential agents.

virus	Primer sequences (5′-3′)	Position
**BPV**	**S GCGCCGCATAAATGTGTCTTGGTG**	**1326∼1349**
	**AS TGTGGGGCTTTCCGCTTTATCTCA**	**1738∼1715**
**FMDV**	**S CGGTGGAAAACTACGGGGGAGAG**	**40∼63**
	**AS TGCGCCGTAGTTGAAGGAGGTTG**	**496∼473**
**BEV**	**S AGGATGATGATTGGCAGATTTTGT**	**372∼395**
	**AS CATGTGGAAGTGTCTTTTGAGGAA**	**708∼685**

BPV: bovine parvovirus; FMDV: foot and mouth disease virus; BEV: bovine enterovirus; S: sense; AS: antisense.

**Table 2:** Primers used for amplifying the complete genome sequence for HY12.

Fragment	primer sequence (5′-3′)	positions
**F1**	**S TTAAAACAGCCTGGGGGTTGTA**	**1∼23**
	**AS GGTGAAATTTGGTAGCATTGCACT**	**1379∼1356**
**F2**	**S CAGTGACACCGACGCAACATC**	**1150∼1170**
	**AS CTAAAGTACATAAGCCCGAGAAAGT**	**3159∼3135**
**F3**	**S TGACGAGAGCATGATCGAGAC**	**2644∼2664**
	**AS CATCTCAGTGAATTTCTTCATCCA**	**4011∼3988**
**F4**	**S AATGGGTTCCGATTCCGTTGTG**	**3886∼3908**
	**AS AGTCAATTCCAGGGAGGTATCAG**	**5632∼5610**
**F5**	**A TCGCTGTCATTCAGAGTGTTTCC**	**5262∼5284**
	**AS GACGGCTTTTTCCTTTCTTGATCTT**	**6485∼6461**
**F6**	**A AATGTGCAAAGAGACATGCCAGAG**	**6455∼6178**
	**AS ACACCCCATCCGGTGGGTGTAT**	**7416∼7395**

S stands for sense; AS refers to antisense.

### Cloning and sequencing

PCR products were analyzed by electrophoresis using 1% agarose gel and cloned to pGEM-T vector (Promega, Madison, WI). Recombinants were confirmed by sequencing (Sangon Biotech, Shanghai). The resulting sequences were analyzed using DNAstar Lasergene software. The nucleotide sequence served as a template for searching homologous sequences through GenBank (www.ncbi.nlm.nih.gov).

### Alignment and phylogenetic analysis

Alignment analysis of multiple sequences was performed using the Clustal W method [32]. The amino acid sequence of HY12 was deduced from the nucleotide sequence. Briefly, the nucleotide sequences of the 5′-UTR, VP1, VP2, VP3 and VP4 were aligned with the corresponding regions of known BEV strains in the GenBank, and phylogenetic analysis was performed by neighbor-joining methods [33].

## Results

### Virus isolation and EM observation

**Figure 1:**
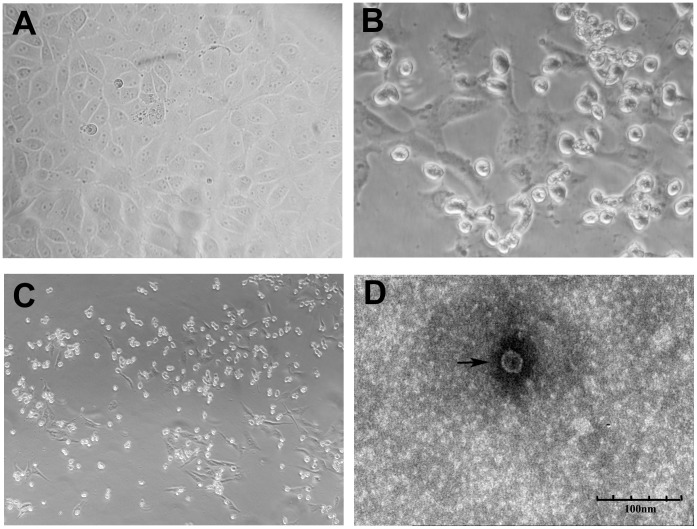
Cytopathic effect of HY12 in cell culture and EM observation. HY12 caused a typical cytopathic effect in MDBK cells after 6–8 h inoculation. Cells became rounded with an increased refraction (B). 24∼48 h post infection, majority of the infected cells detached off the flask (C). The normal MDBK cells were used as negative controls (A). HY12 virus particles observed by electron microscopy to be about 22∼28 nm in diameter as indicated by arrow (D), the scale bar is 100 nm.

To isolate the potential etiological agents, the faecal samples were processed and used to inoculate the MDBK cells, and for electron microscopy observation. After incubating with the inoculum, MDBK cells showed a typical cytopathic effect as early as 6–8 h. Initially, cells became rounded with an increasing refraction. 24∼48 h post inoculation, majority of the infected cells detached off the flask (Fig 1B–C). To rule out the possibility of toxin effect from the sample, the cultures with inoculum were blindly passaged at least 5 generations, and a similar cytopathic effect was observed for each passage, indicating the CPE is the result of pathogen in the inoculum. To determine the potential viruses, the infected cells were frozen and thawed three times and processed for electron microscopy observation. As shown in Fig 1D, virus particles of 22∼28 nm in diameter were observed in infected cells, which was similar to the observation in the faecal sample (not shown). The isolated virus was designated as HY12.

### Virus titration and partial physiochemical properties

To determine the infectivity of HY12, TCID_50_ was determined as previously described [30]. Two days post inoculation, wells (cells) with cytopathic effect were counted. Results from three repeats showed the TCID_50_ for HY12 isolates is 10^−11.68^/0.1 ml. To further characterize the HY12, partial physico-chemical properties, heat-resistant, and acid-resistant experiments were performed. As shown in table 3, TCID_50_ for HY12 isolates has no significant change after treating with either chloroform or ether, suggesting it is a non-enveloped virus. The TCID_50_ has no significant change for treatment at 50°C for 1 h; however viruses completely lost infectivity at 60°C for 1 h, indicating it is sensitive to heat treatment at 60°C. As shown in table 3, HY12 isolates is stable to acid treatment at pH 3.0. Taken together, the above results suggest that HY12 is likely a picornavirus.

**Table 3:** Partial physiochemical properties for HY12 strain.

Treatment		treated groups (TCID50/0.01 ml)	untreated groups (TCID50/0.01 ml)
chloroform		10^−10.1^	10^−10.7^
ether		10^−10.0^	10^−10.2^
Heat-resistant Test(1 h)	50°C	10^−8.4^	10^−10.7^
	60°C	10^−0.0^	10^−10.0^
	70°C	10^−0.0^	10^−10.4^
	80°C	10^−0.0^	10^−10.5^
Acid-resistant(pH)	5.0	10^−8.8^	10^−9.6^
	3.0	10^−9.1^	10^−10.4^

### Molecular characterization and the complete genome sequence of HY12

To characterize the HY12 isolates, the primers for bovine enteroviruses were designed and used to amplify the potential virus genome sequence. Simultaneously, the primers for bovine parvovirus (BPV) and foot and mouth disease virus (FMDV) were used as negative controls. As expected, no fragments for BPV and FMDV were amplified after PCR amplification. However, a fragment with expected size was obtained with the primers for enteroviruses. Further cloning and sequencing this fragment turned out that the fragment consists of a sequence with high homology to a bovine enterovirus strain SL305, confirming HY12′s status as an enterovirus.

To further characterize the virus, the complete nucleotide sequence of HY12 isolates was determined using several pair primers (table 2). After sequencing and assembling the PCR-amplified overlapping fragments, the complete genome sequence of HY12 was revealed to consist of 7416 nucleotides, with a typical picornavirus genome organization including a 5′-UTR, a large single ORF, and a 3′-UTR. The ORF is located between nucleotides 818 and 7348, encoding a polyprotein of 2176 amino acids with a predicted molecular weight of 243 kD. Comparison of the nucleotide sequence revealed that HY12 strain has a similar length of nucleotide sequence for 5′-UTR and 3′-UTR to known BEV strains. The complete nucleotide sequence of HY12 isolates was deposited in GenBank (KF748290).

### Unique amino acid mutations in the capsid protein encoded by HY12

**Figure 2:**
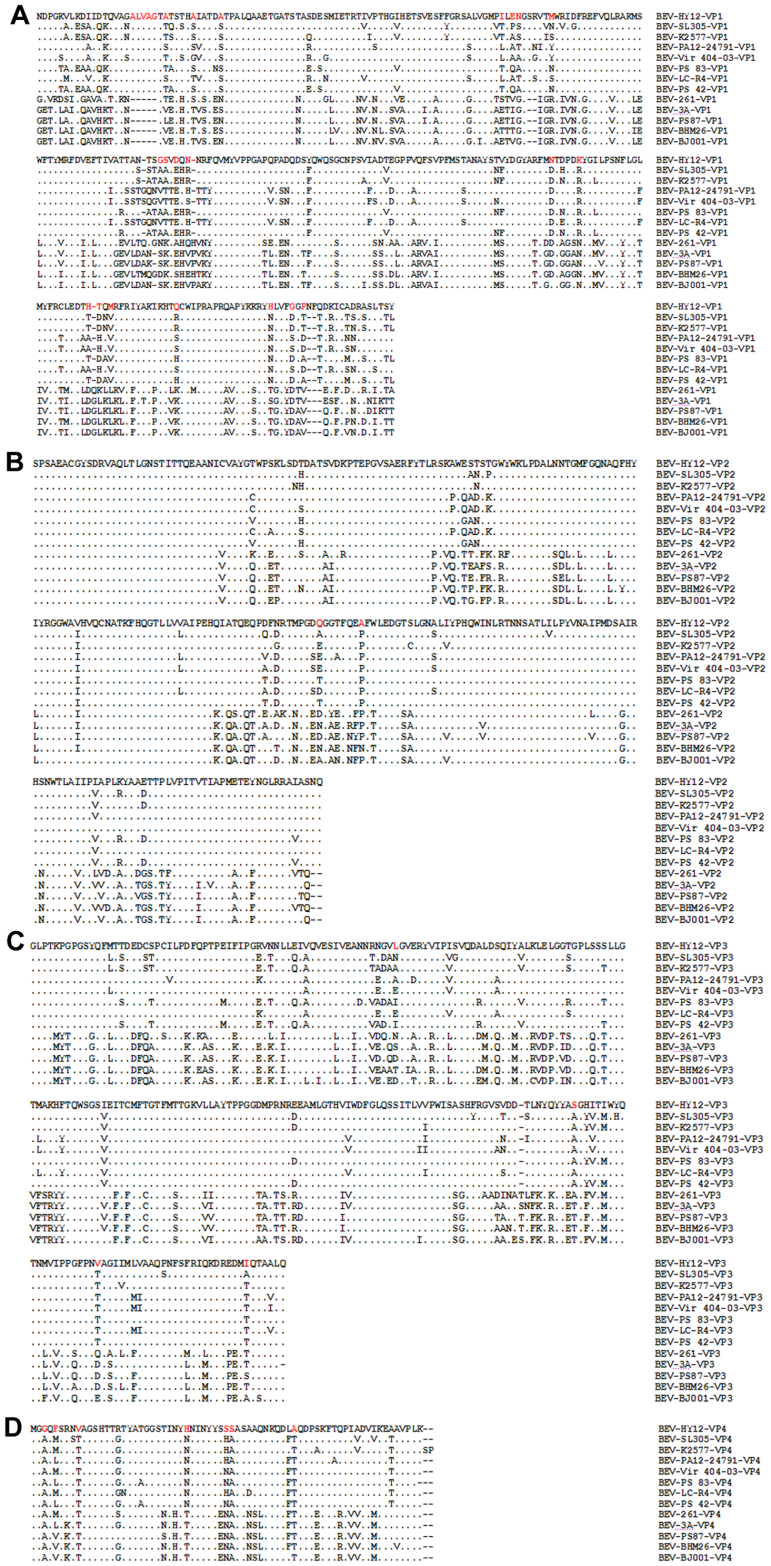
Unique amino acid mutations in the capsid proteins encoded by HY12 isolates. The amino acid sequence of HY12 isolates were deduced from the nucleotide sequence, and was aligned with 12 known BEV strains in the GenBank. Alignment analysis was performed using each HY12-encoded structural protein as a template. Results were shown respectively for VP1 (A), VP2 (B), VP3 (C), and VP4 (D). The identical amino acids were marked with symbol “·”, and different amino acids to HY12 were presented as individual amino acid symbol. The unique mutation for HY12 was highlighted with red color. Deletion of amino acids were marked as “-”.

To analyze the protein encoded by HY12, the deduced amino acid sequence of HY12 was aligned with additional 12 BEV strains. Analysis of deduced amino acid sequence for HY12 revealed a few highly conserved regions including nonstructural proteins 2A, 2B, and P3 in relation to other bovine enterovirus (not shown). Similar to those observed in other enterovirus E, the structural proteins encoded by HY12 contains several relatively conserved regions (Fig. 2). In addition, there are also several variable regions and unique mutations in the capsid proteins, especially in VP1 and VP4. The mutations include an insertion of 5 aa at the N-terminal of VP1; an insertion of 2 aa and a deletion of 1 aa at the middle region of VP1; a unique insertion of 2 aa (NF) at C-terminal region of VP1; and a deletion of 1 aa in the C-terminal for VP3 in relation to the known enterovirus E or F. Furthermore, alignment analysis also revealed rare point mutations within the VP4 for HY12 strain in relation to known enteroviruses E or F (Fig 2).

### HY12 belongs to a novel serotype/genotype in bovine enteroviruses

**Figure 3:**
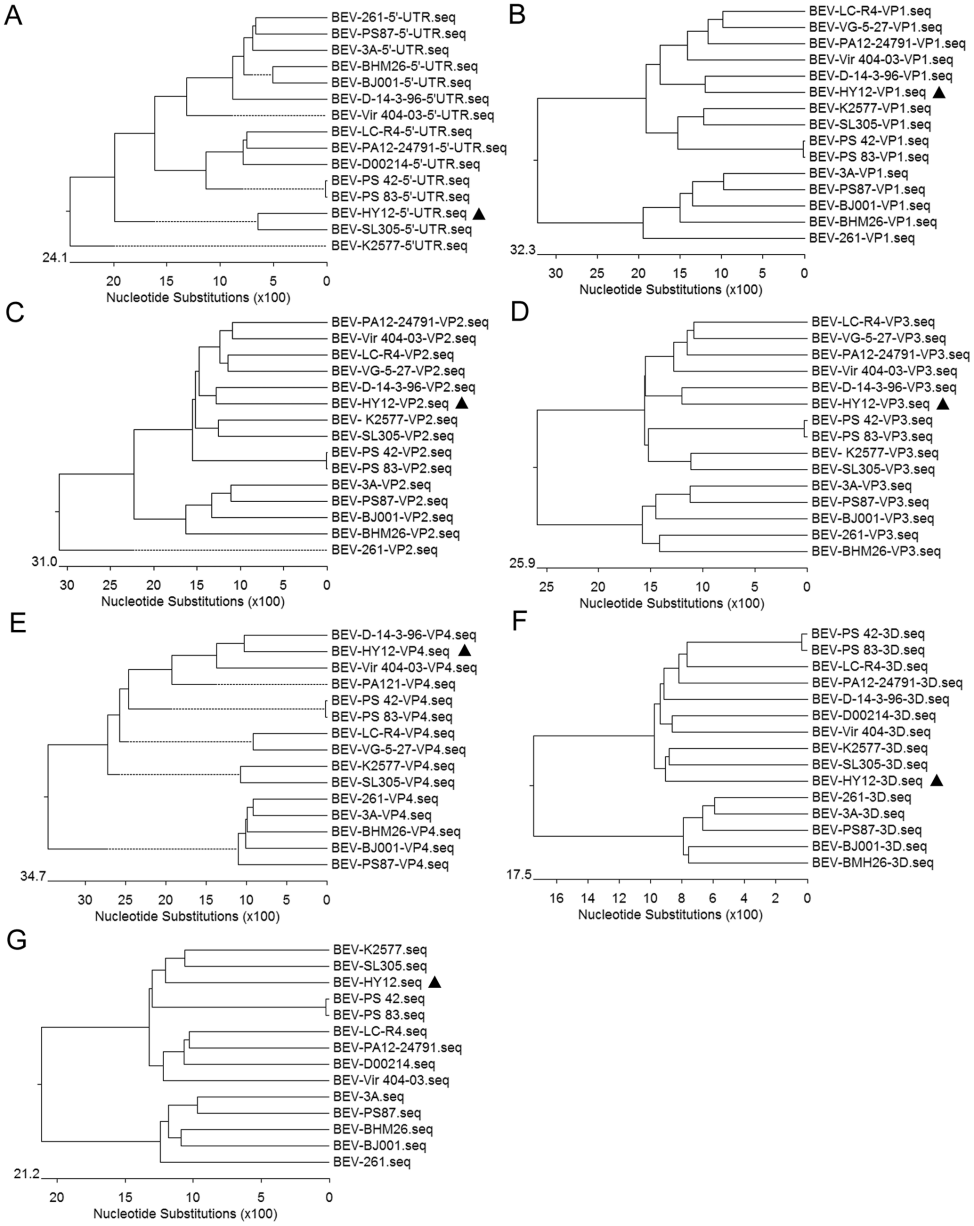
Phylogenetic analysis clustered HY12 strain to a new serotype/genotype within enterovirus E. Phylogenetic tree were generated by neighbor-joining methods by comparing the sequence regions of 5′-UTR, VP1, VP2, VP3, VP4, 3D, and the complete genome sequences for 15 enteroviruses. HY12 strain was placed to the cluster of enteroviruses E after phylogenetic analysis with the all nucleotide sequence regions except 5′-UTR (B–F). The HY12 strain was revealed as a new serotype/genotype (serotype/genotype 3) that only consists of D14/3/96 and HY12 strains in relation to serotype 1 (LC-R4, VG5-27,Vir 404/03) and serotype 2 (SL305, K2577,PS 42, PS 83) enterovirus strains (B–E). When nucleotide sequences for the non-structural proteins 3D and the complete genome sequence were employed, the HY12 were clustered to the same clade most close to SL305 and K2577 within enteroviruses E (F). However, HY12 strain was clustered to neither clade in enteroviruses E nor enterovirus F using the 5′-UTR sequence (A), suggesting an intraserotypic recombination during HY12 evolution. The position of HY12 was highlighted with a triangle.

**Figure 4:**
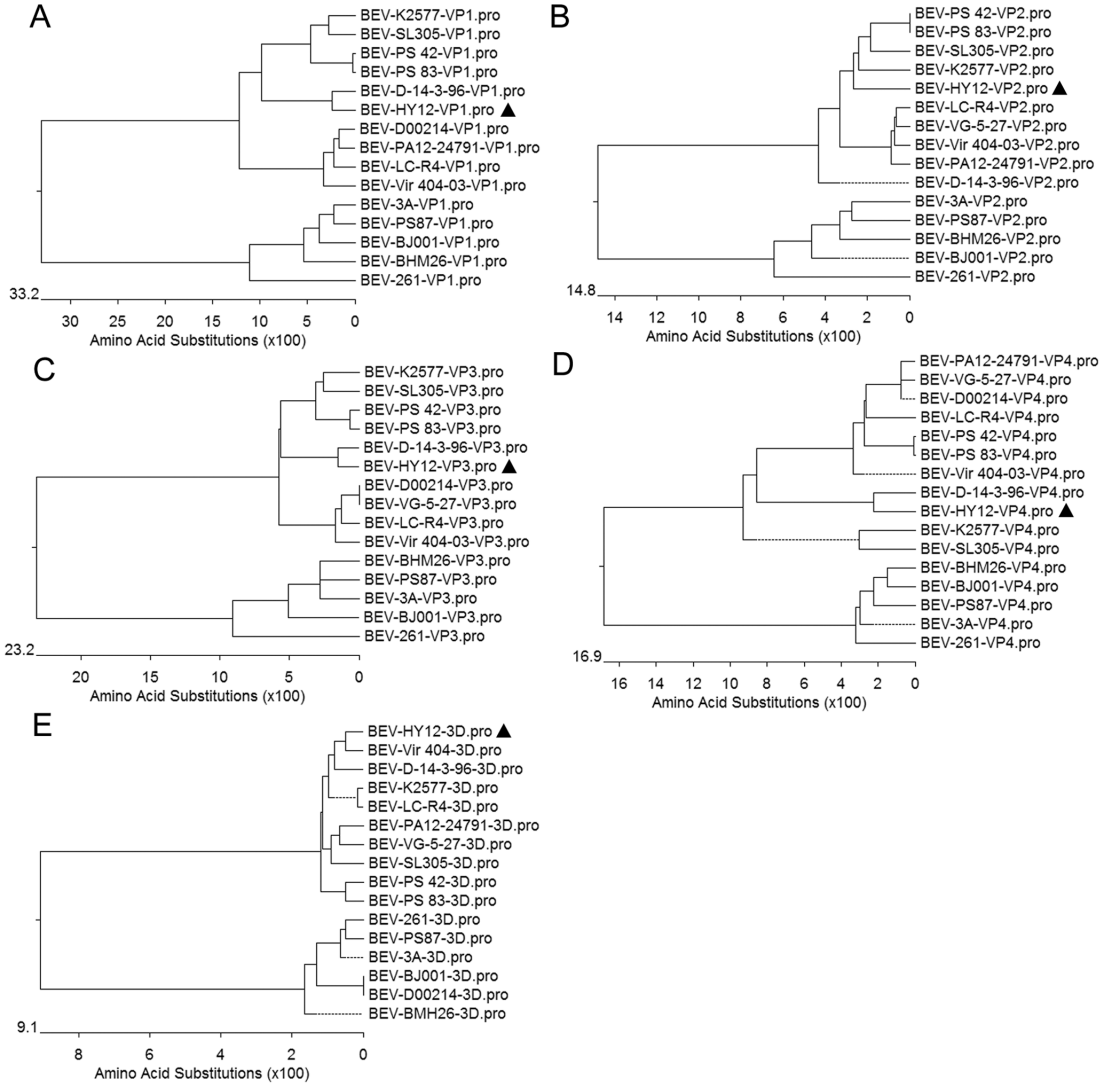
Recombination revealed in the HY12 strain. Neighbor-joining trees of the structural proteins VP1-VP4, and the non-structural protein 3D of 15 enteroviruses were compared. When amino acid sequences for VP1, VP3, and VP4 were used to generate phylogenetic tree, similar patterns were observed as those in Fig 3B, 3D, and 3E, indicating a interserotype recombination for the HY12 strain. Like the observation in Fig 3A, F and G, the HY12 was clustered closely to K2577, SL305, PS 42 and PS 83 strains, an indication of complex interserotypic and intraserotypic recombination in the evolution for HY12. The positions of HY12 were highlighted with a triangle.

To define the relationship of HY12 with other enteroviruses, the nucleotide sequences of the 5′-UTR, VP1, VP2, VP3, VP4, 3D, the complete genome sequence of HY12 were aligned with the corresponding regions of known BEV strains, and phylogenetic trees were generated by neighbor-joining methods. As shown in Fig 3, when the nucleotide sequences encoding the structural proteins (VP1,VP2, VP3,VP4) for HY12 were used to generate the phylogenetic tree, the HY12 strain was clustered to enteroviruses E, but was neither in serotype/genotype 1 (LC-R4, VG5-27,Vir 404/03) nor serotype/genotype 2 (SL305, K2577,PS 42, PS 83), it belongs to a new serotype/genotype consisting of the D14/3/96 and HY12 (Fig 3B-E). We named it as serotype/genotype 3. It is interesting to note when nucleotide sequences for the non-structural proteins (3D) and the complete genome sequence were employed, the HY12 were clustered to the same clade most close to SL305 and K2577 within enteroviruses E (Fig 3A, F, and G). However, when the 5′-UTR sequence was aligned with other enteroviruses, the sequence identity of HY12 was 71.7∼86.7% with other enteroviruses, and phylogenetic analysis showed the HY12 is neither in the clade for enteroviruses E nor in enterovirus F, it is in a clade with SL305, close to K2577, another separate clade (Fig 3A). As shown in Fig 4, similar expected patterns were revealed when amino acid sequences for VP1, VP3, and VP4 were used to generate phylogenetic tree. Like the observation in Fig 3A, F and G, the HY12 was clustered closely to K2577, SL305, PS 42 and PS 83 (Fig 4B). The above results indicated a complex interserotypic and intraserotypic recombination in the evolution for HY12.

## Discussion

In this study, we reported the isolation of an enterovirus HY12 strain from a cattle farm with an unknown disease of high morbidity and mortality. Sequencing results demonstrated that HY12 is a new isolates within enterovirus E. As far as we know, this is the first report of enterovirus E in China. Enteroviruses E and F both belong to the genus *enterovirus* that are etiologically associated with bovine enterovirus infections with clinical signs varying from respiratory diseases to enteritic, reproductive disease and infertility [2], [3], [4], [5], [6], [7], [8], [9], [10], [11], [12]. Although there are reports neglecting the pathogenicity of BEVs, increasing reports for isolation of BEVs from respiratory and diarrheic cattle suggest the relatedness of BEV with those diseases. Our detection and isolation of bovine enterovirus HY12 in a cattle farm with severe respiratory and enteric diseases clearly indicate that HY12 might play a critical role in this outbreak as etiological agents.

The classification of bovine enteroviruses undergoes an evolution since their initial discovery. Zell reported a molecular-based BEV classification system based on virus genetic variability and molecular difference, and demonstrated a congruence of this molecular-based classification with previous classification methods [29]. Based on the generally accepted definition for picornavirus species and serotype, BEVs were classified into enterovirus E (BEV-A) and enterovirus F (BEV-B) by comparing sequences from 5′-UTR, 3′UTR and capsid protein region, where a different serotypes were further divided for either enterovirus E or enterovirus F [27], [28], [29]. It is generally accepted that picornavirus serotypes are molecularly defined by the diversity of the capsid proteins, while the enterovirus species were determined by the less diverse non-structural protein regions. The percentage of sequence identity was used for species/serotypes definition, where a range from 50% to 55% for heterologous species, 70% to 85 % for heterologous serotypes/homologous species, and greater than 90 % for homologous serotypes [29]. According to this criteria, we defined HY12 enterovirus isolates as enterovirus E since the amino acid sequence identity of VP1 encoded by HY12 is only ranged from 53% to 55% with previously identified BEV-B viruses such as BEV strains PS87, 3A, 261, and two newly strains isolated in China (BHM26, and BJ001). The finding that VP1 of HY12 is about 80-83% with those previously defined BEV-A suggests HY12 is a heterogeneous serotype within enteroviruses E. Moreover, phylogenetic analysis clearly showed that HY12 isolates belongs to neither serotype/genotype 1 (SL305, K2577 PS42, PS83) nor serotype/genotype 2 (LC-R4, Vir 404-03 PA12-24791); it belongs to a new serotype/genotype. The results that HY12 is phylogenetically clustered with D 14/3/96 strain [29], a strain reported to be difficult in serotype/genotype typing further support the classification of HY12 to new serotype/genotype within enterovirus E.

Interserotypic and intraserotypic recombination have been defined previously in poliovirus, echovirus and enteroviruses [1], [34], [35], [36], [37]. The incongruence between phylogenies of different genome regions is considered as an indication of recombination events in enteroviruses [29], [38]. Phylogenetic analyses of the 5′-UTR, VP1, VP2, VP3, VP4, and 3D of HY12 demonstrated the incongruence between the phylogenies, indicating a complex interserotypic and intraserotypic recombination in the evolution of HY12 isolates.

In conclusion, this study reports the isolation and characterization of a novel enterovirus HY12 strain from a severe outbreak characterized with respiratory and enteric disease in a cattle farm with high mortality and morbidity in China, and provides the molecular evidence for defining HY12 strain as a new serotype/genotype 3 within enterovirus E.

## References

[1] King AMQ, Adams MJ, Carsten EB, Lefkowitz E (2012) Virus Taxonomy. Ninth Report of the International Committee for the Taxonomy of Viruses. San Diego: Academic Press. 855–880 p.

[2] Moll T, Davis AD (1959) Isolation and characterization of cytopathogenic enterovirus from cattle with respiratory disease. Am J Vet Res 20: 27–32. 14423386

[3] Straub OC, Boehm HO (1964) Enterovirus as Cause of Bovine Vaginitis. Arch Gesamte Virusforsch 14: 272–275. 14141587 10.1007/BF01555099

[4] Buerki F (1962) Studies on bovine Enterovirus. 3. Epizootologic survey. Pathol Microbiol (Basel) 25: 789–795. 14016650

[5] Straub OC, Kielwein G (1965) Bovine enteroviruses as causative agents of mastitis. Berl Munch Tierarztl Wochenschr 78: 386–389. 5864684

[6] Rovozzo GC, Luginbuhl RE, Helmboldt CF (1965) Bovine Enteric Cytopathogenic Viruses. I. Characteristics of Three Prototype Strains. Cornell Vet 55: 121–130. 14284441

[7] Van der Maaten MJ, Packer RA (1967) Isolation and characterization of bovine enteric viruses. Am J Vet Res 28: 677–684. 4292082

[8] Buczek J (1970) Further characterization of bovine enteroviruses isolated in Poland. Brief report. Arch Gesamte Virusforsch 30: 408–410. 4318627 10.1007/BF01258371

[9] Ide PR (1970) Developments in veterinary science. The etiology of enzootic pneumonia of calves. Can Vet J 11: 194–202. 4321488 PMC1695118

[10] Dunne HW, Ajinkya SM, Bubash GR, Griel LC Jr (1973) Parainfluenza-3 and bovine enteroviruses as possible important causative factors in bovine abortion. Am J Vet Res 34: 1121–1126. 4355769

[11] Weldon SL, Blue JL, Wooley RE, Lukert PD (1979) Isolation of picornavirus from feces and semen from an infertile bull. J Am Vet Med Assoc 174: 168–169. 221445

[12] Li Y, Chang J, Wang Q, Yu L (2012) Isolation of two Chinese bovine enteroviruses and sequence analysis of their complete genomes. Arch Virol 157: 2369–2375. 22851010 10.1007/s00705-012-1424-6 PMC7087101

[13] Zheng T (2007) Characterisation of two enteroviruses isolated from Australian brushtail possums (Trichosurus vulpecula) in New Zealand. Arch Virol 152: 191–198. 16906477 10.1007/s00705-006-0838-4 PMC7086802

[14] McCarthy FM, Smith GA, Mattick JS (1999) Molecular characterisation of Australian bovine enteroviruses. Vet Microbiol 68: 71–81. 10501163 10.1016/s0378-1135(99)00062-0

[15] McFerran JB (1958) ECBO viruses of cattle. Vet Rec 70: 999.

[16] Ley V, Higgins J, Fayer R (2002) Bovine enteroviruses as indicators of fecal contamination. Appl Environ Microbiol 68: 3455–3461. 12089028 10.1128/AEM.68.7.3455-3461.2002 PMC126779

[17] Dubois E, Merle G, Roquier C, Trompette AL, Le Guyader F, et al (2004) Diversity of enterovirus sequences detected in oysters by RT-heminested PCR. Int J Food Microbiol 92: 35–43. 15033266 10.1016/j.ijfoodmicro.2003.06.001

[18] Fong TT, Griffin DW, Lipp EK (2005) Molecular assays for targeting human and bovine enteric viruses in coastal waters and their application for library-independent source tracking. Appl Environ Microbiol 71: 2070–2078. 15812040 10.1128/AEM.71.4.2070-2078.2005 PMC1082535

[19] Jimenez-Clavero MA, Escribano-Romero E, Mansilla C, Gomez N, Cordoba L, et al (2005) Survey of bovine enterovirus in biological and environmental samples by a highly sensitive real-time reverse transcription-PCR. Appl Environ Microbiol 71: 3536–3543. 16000759 10.1128/AEM.71.7.3536-3543.2005 PMC1168977

[20] Nollens HH, Rivera R, Palacios G, Wellehan JF, Saliki JT, et al (2009) New recognition of Enterovirus infections in bottlenose dolphins (Tursiops truncatus). Vet Microbiol 139: 170–175. 19581059 10.1016/j.vetmic.2009.05.010 PMC4310689

[21] Blas-Machado U, Saliki JT, Boileau MJ, Goens SD, Caseltine SL, et al (2007) Fatal ulcerative and hemorrhagic typhlocolitis in a pregnant heifer associated with natural bovine enterovirus type-1 infection. Vet Pathol 44: 110–115. 17197635 10.1354/vp.44-1-110

[22] Blas-Machado U, Saliki JT, Sanchez S, Brown CC, Zhang J, et al (2011) Pathogenesis of a bovine enterovirus-1 isolate in experimentally infected calves. Vet Pathol 48: 1075–1084. 21245281 10.1177/0300985810395728

[23] McClenahan SD, Scherba G, Borst L, Fredrickson RL, Krause PR, et al (2013) Discovery of a bovine enterovirus in alpaca. PLoS One 8: e68777. 23950875 10.1371/journal.pone.0068777 PMC3741315

[24] Cho YI, Han JI, Wang C, Cooper V, Schwartz K, et al (2013) Case-control study of microbiological etiology associated with calf diarrhea. Vet Microbiol 166: 375–385. 23886509 10.1016/j.vetmic.2013.07.001 PMC7117237

[25] Dunne HW, Huang CM, Lin WJ (1974) Bovine enteroviruses in the calf: an attempt at serologic, biologic, and pathologic classification. J Am Vet Med Assoc 164: 290–294. 4359896

[26] Knowles NJ, Barnett IT (1985) A serological classification of bovine enteroviruses. Arch Virol 83: 141–155. 2578783 10.1007/BF01309912

[27] Zell R, Stelzner A (1997) Application of genome sequence information to the classification of bovine enteroviruses: the importance of 5′- and 3′-nontranslated regions. Virus Res 51: 213–229. 9498619 10.1016/s0168-1702(97)00096-8

[28] Zell R, Sidigi K, Henke A, Schmidt-Brauns J, Hoey E, et al (1999) Functional features of the bovine enterovirus 5′-non-translated region. J Gen Virol 80 (Pt 9): 2299–2309. 10.1099/0022-1317-80-9-2299 10501480

[29] Zell R, Krumbholz A, Dauber M, Hoey E, Wutzler P (2006) Molecular-based reclassification of the bovine enteroviruses. J Gen Virol 87: 375–385. 16432025 10.1099/vir.0.81298-0

[30] Reed LJ, Muench H (1938) A simple method of estimating fifty percent endpoints. Am J Hygiene: 493–497.

[31] Wang XP, Zhang YJ, Deng JH, Pan HY, Zhou FC, et al (2001) Characterization of the promoter region of the viral interferon regulatory factor encoded by Kaposi's sarcoma-associated herpesvirus. Oncogene 20: 523–530. 11313983 10.1038/sj.onc.1204115

[32] Thompson JD, Higgins DG, Gibson TJ (1994) CLUSTAL W: improving the sensitivity of progressive multiple sequence alignment through sequence weighting, position-specific gap penalties and weight matrix choice. Nucleic Acids Res 22: 4673–4680. 7984417 10.1093/nar/22.22.4673 PMC308517

[33] Saitou N, Nei M (1987) The neighbor-joining method: a new method for reconstructing phylogenetic trees. Mol Biol Evol: 406–425. 10.1093/oxfordjournals.molbev.a040454 3447015

[34] Kim H, Kim K, Kim DW, Jung HD, Min Cheong H, et al (2013) Identification of Recombinant Human Rhinovirus A and C in Circulating Strains from Upper and Lower Respiratory Infections. PLoS One 8: e68081. 23826363 10.1371/journal.pone.0068081 PMC3695095

[35] Yozwiak NL, Skewes-Cox P, Gordon A, Saborio S, Kuan G, et al (2010) Human enterovirus 109: a novel interspecies recombinant enterovirus isolated from a case of acute pediatric respiratory illness in Nicaragua. J Virol 84: 9047–9058. 20592079 10.1128/JVI.00698-10 PMC2937614

[36] McIntyre CL, McWilliam Leitch EC, Savolainen-Kopra C, Hovi T, Simmonds P (2010) Analysis of genetic diversity and sites of recombination in human rhinovirus species C. J Virol 84: 10297–10310. 20668080 10.1128/JVI.00962-10 PMC2937769

[37] Boros A, Pankovics P, Knowles NJ, Reuter G (2012) Natural interspecies recombinant bovine/porcine enterovirus in sheep. J Gen Virol 93: 1941–1951. 22647375 10.1099/vir.0.041335-0

[38] Smura T, Blomqvist S, Paananen A, Vuorinen T, Sobotova Z, et al (2007) Enterovirus surveillance reveals proposed new serotypes and provides new insight into enterovirus 5′-untranslated region evolution. J Gen Virol 88: 2520–2526. 17698662 10.1099/vir.0.82866-0

